# The use of intact fish skin grafts in the treatment of acute complicated bite wounds: a case report

**DOI:** 10.1093/jscr/rjag197

**Published:** 2026-03-26

**Authors:** Ivan Adamovic, Hans-Dieter Mäder, Alexander Golowan, Kostas Michael, Thibika Sivakumar, Jörg Lucian, Lukas Krähenbühl

**Affiliations:** Department of Surgery, ASANA Spital Leuggern, 5316 Leuggern, Switzerland; Department of Surgery, ASANA Spital Leuggern, 5316 Leuggern, Switzerland; Department of Surgery, ASANA Spital Leuggern, 5316 Leuggern, Switzerland; Department of Surgery, ASANA Spital Leuggern, 5316 Leuggern, Switzerland; Department of Surgery, ASANA Spital Leuggern, 5316 Leuggern, Switzerland; Department of Surgery, ASANA Spital Leuggern, 5316 Leuggern, Switzerland; Department of Surgery, ASANA Spital Leuggern, 5316 Leuggern, Switzerland

**Keywords:** fish skin, grafts, complicated wounds, bite wounds

## Abstract

The management of complex bite wounds, particularly in patients with comorbidities, presents a significant clinical challenge due to the high risk of infection and prolonged healing times. This case report highlights the successful use of intact fish skin grafts as a novel treatment modality for a primary contaminated bite wound at the patient with comorbidities such as diabetes mellitus and rheumatoid artritis with cortisone use. A 54-year-old European female patient with a medical history of rheumatoid arthritis and type 2 diabetes mellitus presented with a large, full-thickness dog bite wound on her right lower leg. Surgical debridement and broad-spectrum antibiotic administration, an intact Kerecis fish skin graft was applied and secured. The application of an intact fish skin xenograft, combined with surgical debridement and appropriate antibiotic therapy, proved to be a highly effective treatment for a complex bite wound in an immunocompromised patient.

## Introduction

Existing evidence shows that acellular fish skin grafts AFSGs accelerate wound healing, reduce pain, prevent antibiotic administration, and cause no autoimmune reactions [1, 2].

A search of major medical databases to our knowledge does not reveal any dedicated case reports, case series, observational studies, or clinical trials on the use of Kerecis for human bite wounds. However, some research on primarily contaminated wounds suggests that Kerecis fish skin xenograft products, which have been introduced to treat diabetic wounds, traumatic wounds, partial-thickness burns, and necrotic wounds, may be applicable [3, 4].

Microorganisms found in bite wounds from cats and dogs are often a mix of bacteria from the animal's oral flora and the human's skin flora. One microorganism stands out as the most common and clinically significant pathogen, especially in cat and dog bites: *Pasteurella multocida*. It causes a rapidly progressing infection with intense pain, redness, and swelling, often appearing within 24 hours of the bite [5]. While *P. multocida* typically causes an acute infection, it can lead to a prolonged, chronic wound in patients with underlying conditions, especially by immunocompromised patients [6].

Early identification and surgical debridement and antibiotics application are critical for managing this kind of injury and its associated complications. Following the removal of ischemic and necrotic tissue, reconstructing the substantial soft-tissue defects is a necessity. Such defects may require the use of skin flaps or split-thickness grafts, while others may necessitate a free-tissue transfer [7].

The Kerecis™ fish skin xenograft is a product derived from minimally processed North Atlantic Cod, which allows it to act similarly to human skin. The porous structure of Kerecis prevents fluids from collecting in potential pockets of the bite wound, which avoids an additional risk of abscess formation or the spread of infection [8].

## Case report

**Figure 1:**
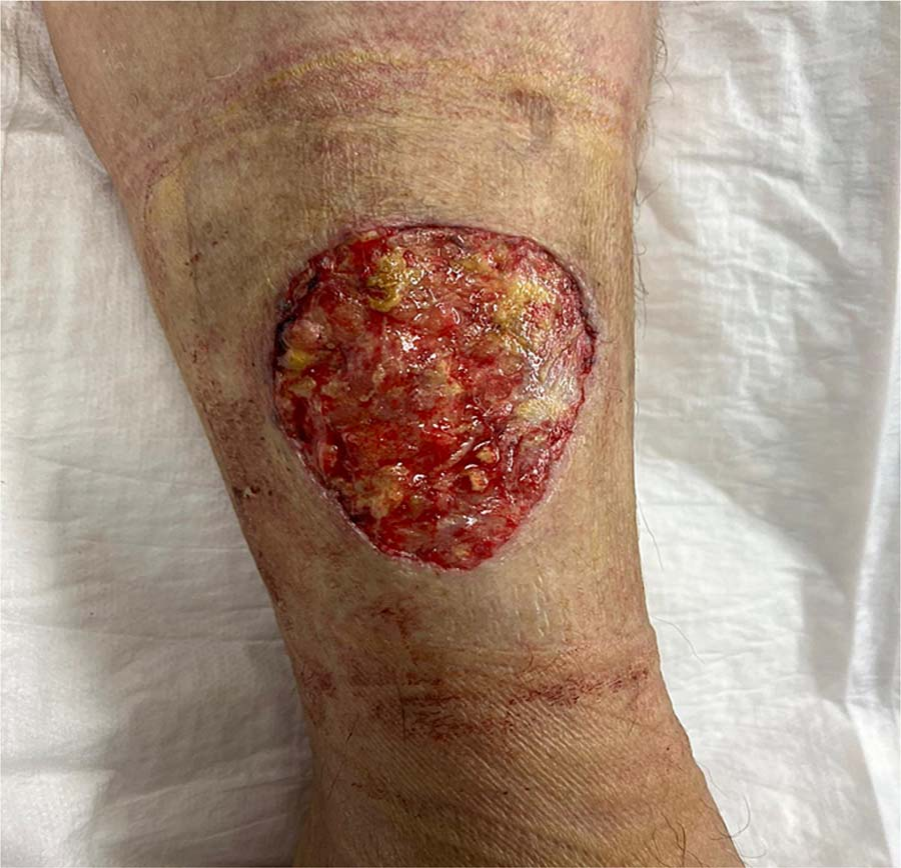
Wound-large skin and subcutaneous defect on the distal-lateral aspect of her right lower leg.

A 54-year-old European female patient presented immediately after sustaining a dog bite injury from own dog on 28 September 2024. She presented with an open wound and a large skin and subcutaneous defect (6 cm wide × 6 cm long and 0.6 cm deep) on the distal-lateral aspect of her right lower leg. Her medical history was significant for rheumatoid arthritis (using corticosteroids) and type 2 diabetes mellitus, which are both known to cause delayed wound healing (Fig. 1).

Clinical assessment showed absence of clinical signs of infection. The patient's history included a tendency for prolonged wound healing after injuries and the formation of scar tissue.

**Figure 2:**
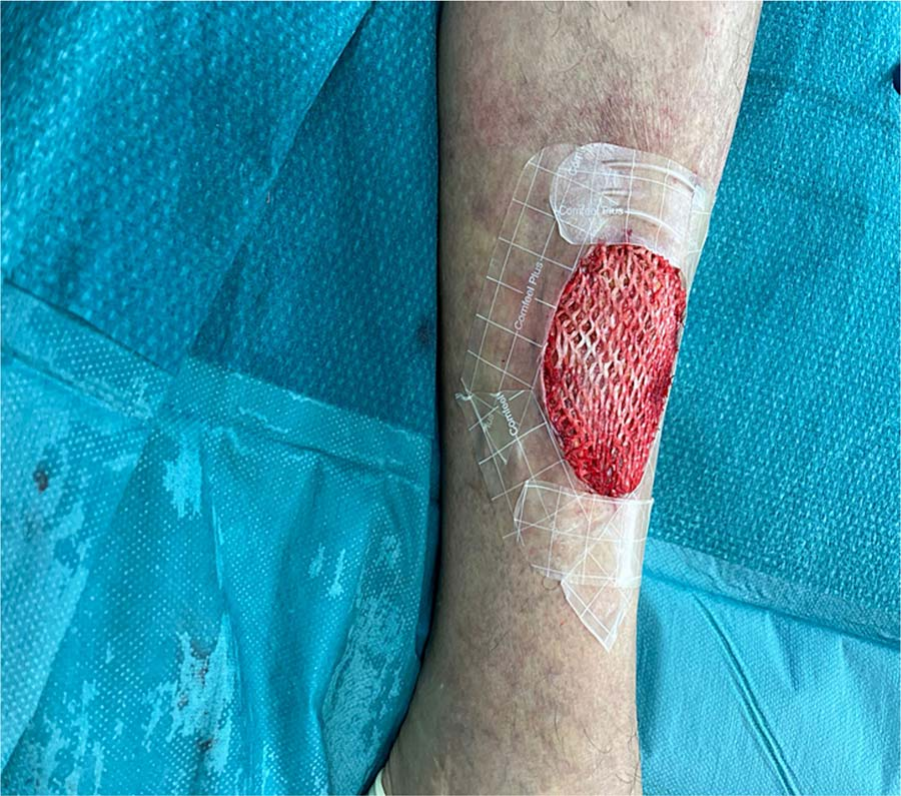
Application of Kerecis graft.

**Figure 3:**
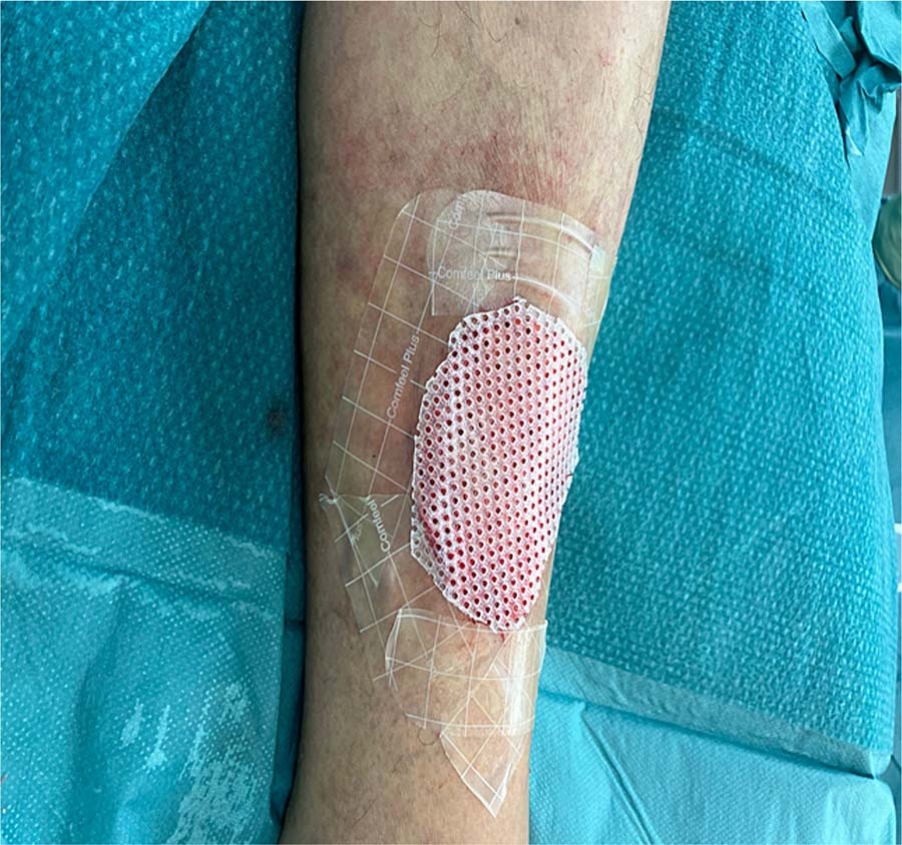
Application of a Mepitel spacer grid.

Given her history of prolonged wound healing our treatment plan included surgical debridement in the operating theatre to remove all ischemic and non-viable tissue. This was followed by the application of a Kerecis fish skin graft to cover the defect (Fig. 2). The graft was fixated to the intact skin edges with Monocryl 4–0 sutures to prevent migration. Application of a Mepitel spacer grid protected the graft after dressing change (Fig. 3).

Microbiology results isolated only *P. multocida*, which was found to be sensitive to amoxicillin-clavulanic acid. The studies showed that the best treatment for animal bites with suspected *Pasteurella* spp usually entails a combination of amoxicillin and the β-lactamase inhibitor clavulanic acid [9]. For most superficial bite wound infections without deep tissue involvement, a 7- to 10-day course is generally sufficient. Drug resistance in Pasteurella spp human infections has rarely been reported in the literature [10].

**Figure 4:**
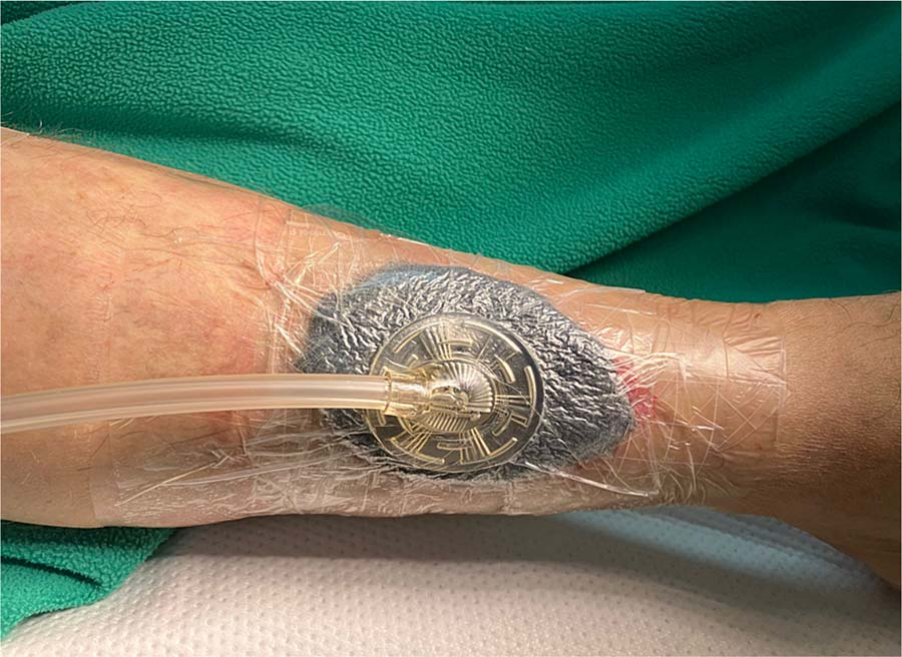
Application of a NPWT.

The wound was then managed with negative pressure wound therapy (NPWT) for 3 days to optimize the wound bed and promote healing (Fig. 4). Continuous negative pressure of 125 mmHg was applied.

**Figure 5:**
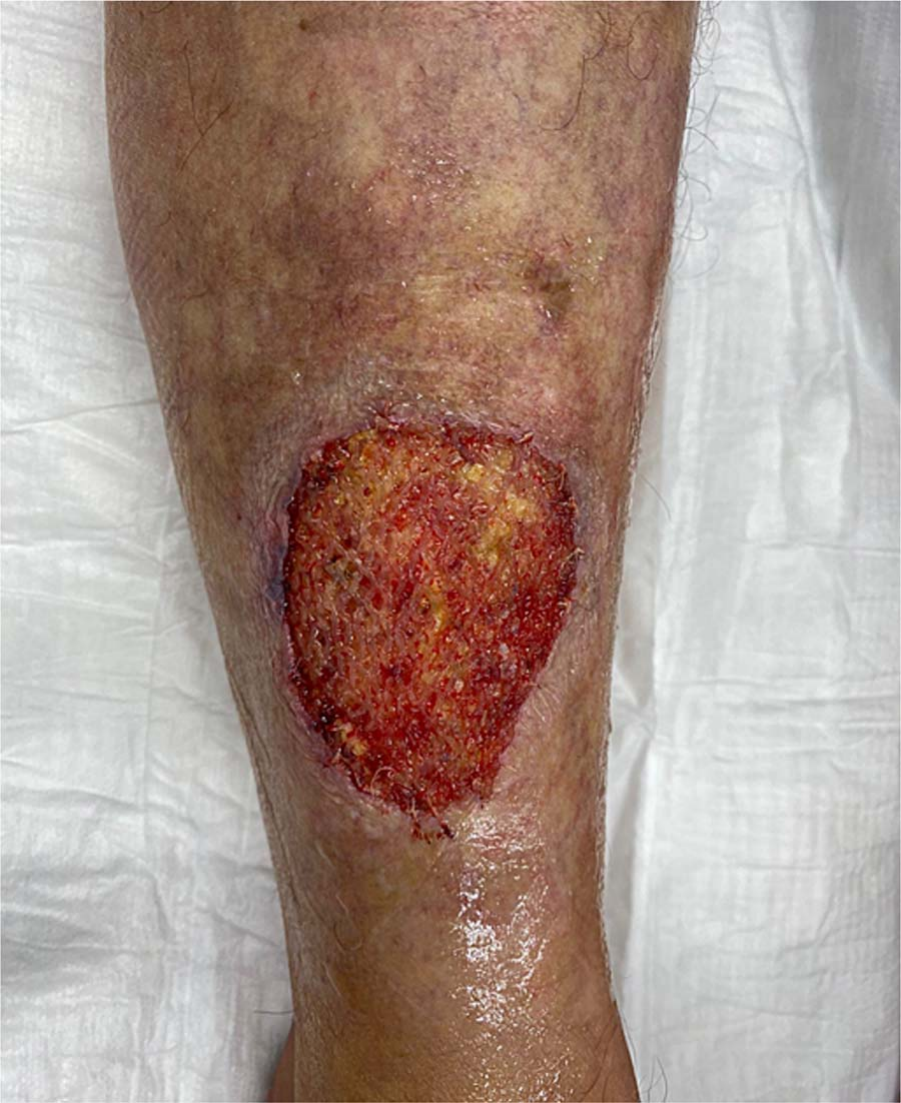
Wound assessment three days postoperatively, following removal of the VAC dressing: Showing a partially integrated Kerecis fish skin graft with beginning granulation and without any signs of infection.

After VAC removal, we discharged the patient on the third postoperative day (Fig. 5).

**Figure 6:**
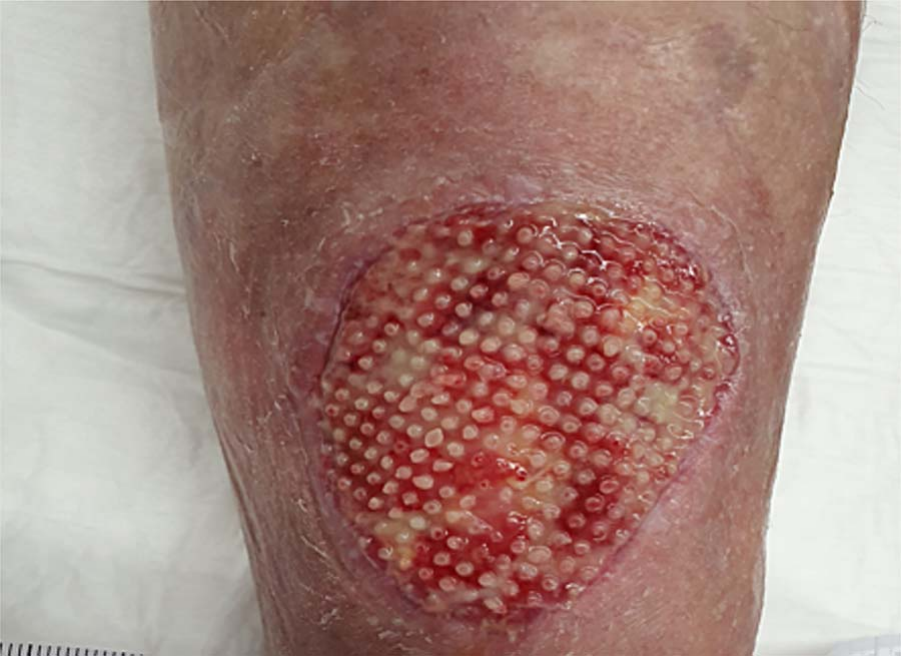
Four weeks postoperatively.

Follow-up inspections have been done twice a week (Monday and Thursday). Following the removal of the previous dressing, the wound was cleaned and disinfected with hypochlorous acid. A non-adherent spacer grid–Mepitel, was then applied to the wound bed to protect the new tissue. This was followed by the application of a superabsorbent pad to manage any exudate. Figures 6 through 9 illustrate the progression of the wound until complete healing:

**Figure 7:**
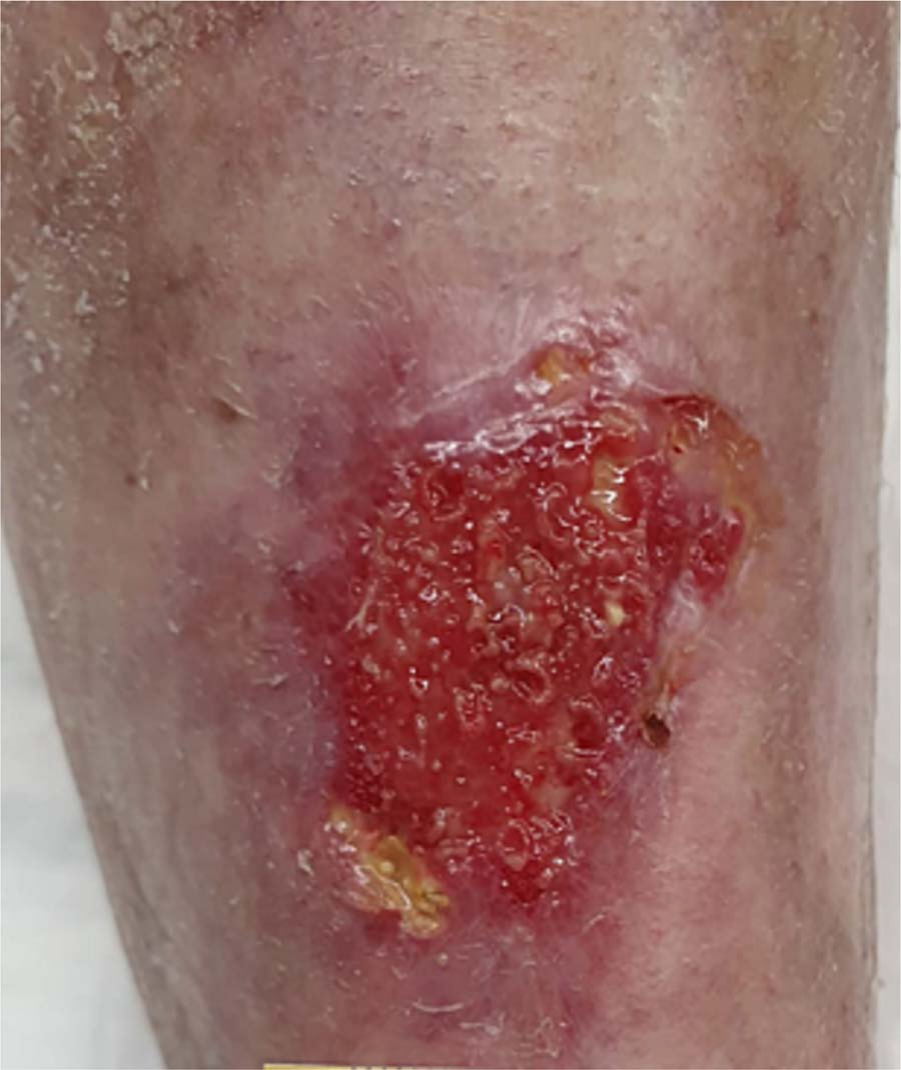
Six weeks postoperatively.

**Figure 8:**
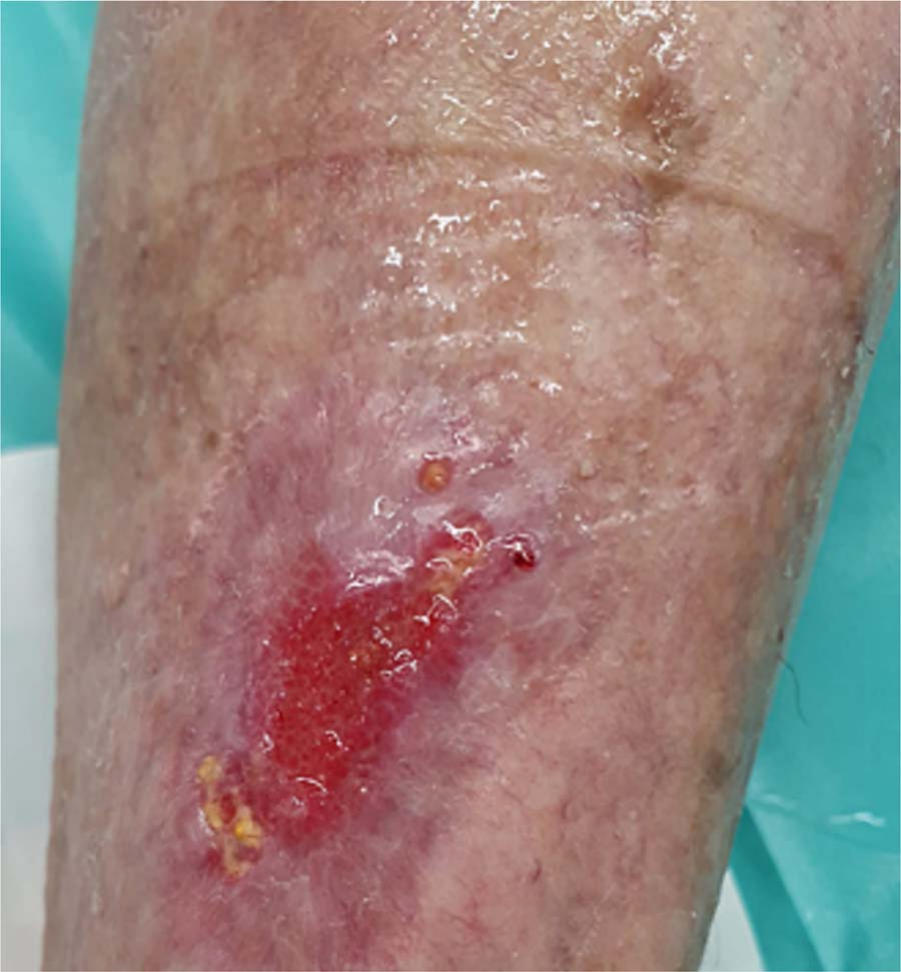
Eight weeks postoperatively.

**Figure 9:**
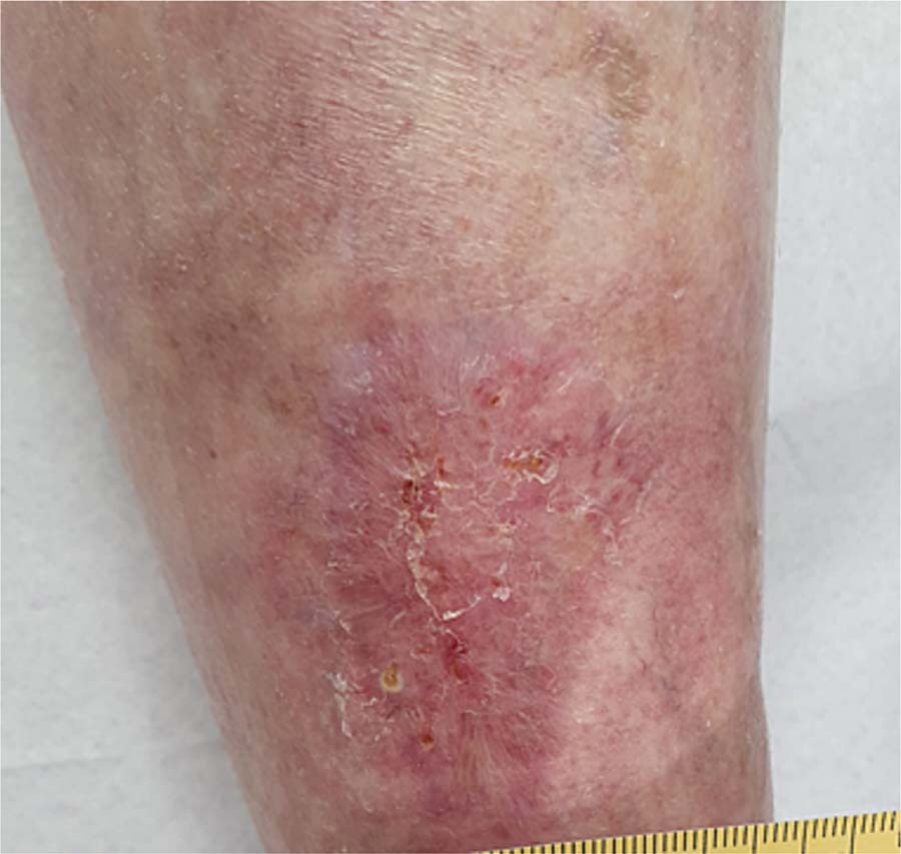
Wound closure after 10 weeks.

We achieved wound closure in 10 weeks. After a 6-month follow-up, there was no new re-opening of the wound, and the skin and scar quality was much better and more elastic than the rest of the parchment skin. Important dates and times in this case are presented in Table 1 as a timeline.

**Table 1:** Case report timeline

Date/Timeframe	Event
28 September 2024 (Admission)	Patient presented immediately after dog bite
Day 1 (Surgery)	Surgical debridement performed in the operating theatre and Kerecis fish skin graft applied.
Day 3 (Post-Op)	NPWT removed and patient discharged. Wound check performed
Follow-up inspections twice a week (Monday and Thursday) from Day 4 till Day 28.	Routine dressing changes and wound care by the same team.
4 Weeks Post-Op	Wound check with illustration shows continued healing and granulation.
6 Weeks Post-Op	Wound check with illustration shows continued healing and granulation.
8 Weeks Post-Op	Wound check with illustration shows continued healing and granulation.
10 Weeks Post-Op	Final assessment. Wound closure achieved. The patient was completely mobile without pain.
6-Month Follow-up	Final evaluation. No new re-opening of the wound; scar quality was noted to be much better and more elastic than the surrounding parchment skin

## Discussion

Full-thickness wounds in patients with comorbidities and infection can take up to 24 months to heal. An established infection, as is often caused by *P. multocida*, significantly delays healing. Pre-existing conditions such as diabetes mellitus or rheumatoid arthritis are known to impair wound healing as well [11, 12]. According to these findings, we observed that the application of AFSGs-Kerecis accelerated wound healing to only 10 weeks. The healing progressed steadily without any deterioration.

Scar tissue tensile strength will only reach 80%, compared to the original tissue [13]. In our case, the quality of the scar tissue was clinically very good and more elastic than the existing parchment skin. The tissue's tensile strength was 100%, which is 20% greater than the average outcome.

In our experience the consistent application of follow-up and wound care by the same team of experts is of great importance. This continuity ensures a unified treatment strategy and accurate assessment of the wound's progress, which are critical for optimal healing outcomes.

In the existing literature to our knowledge, we did not find any supportive paper on the use of fish skin grafts for human bite wounds, with the exception of one case study on animals [14].

Using the Kerecis graft as a proactive measure, even in primary bite wounds without immediate signs of infection, is a key takeaway. This approach aims to prevent complications from developing, rather than treating them after they occur.

The findings of this case should encourage further research. A controlled study comparing outcomes of traditional wound care with Kerecis grafts in similar patient populations would provide more robust evidence and help establish it as a standard of care.

## Supplementary Material

CARE_-_checklist_rjag197

## Data Availability

The dataset supporting the conclusions of this article is available from the corresponding author upon reasonable request.
