# Supplementary material for: The use of intact fish skin grafts in the treatment of acute complicated bite wounds: a case report
Source: J Surg Case Rep. 2026 Mar 26;2026(3):rjag197. doi: 10.1093/jscr/rjag197 (PMC13019289; doi:10.1093/jscr/rjag197)
Supplement: CARE_-_checklist_rjag197 [file care_-_checklist_rjag197.docx]

**CARE  Checklist  of  information for case report**

**01.Title:** «The Use of Intact Fish Skin Grafts in the Treatment of Acute Complicated Bite Wounds:

a case report»

**02. Key Words:** Fish skin grafts, bite wounds, complicated wounds

**Abstract**

**3a Introduction:** What is unique about this case and what does it add to the scientific literature?

The management of complex bite wounds, particularly in patients with comorbidities, presents a significant clinical challenge due to the high risk of infection and prolonged healing times. While the use of cellular, acellular, and matrix-like products (CAMPs) has shown promise in other wound types, there is a lack of high-level evidence supporting their use in human bite wounds. This case report highlights the successful use of intact fish skin grafts as a novel treatment modality for a primary contaminated bite wound at the patient with comorbidities such as diabetes mellitus and rheumatoid artritis with cortisone use.

**3b Main symptoms and/or important clinical findings:**

A 54-year-old female with a medical history of rheumatoid arthritis and type 2 diabetes mellitus presented with an acute, contaminated dog bite wound on her lower leg, measuring 6 cm x 6 cm x 0.6 cm with significant skin and soft tissue defect.

**3c The main diagnoses, therapeutic interventions, and outcomes:**

The Main Diagnoses, Therapeutic Interventions, and Outcomes: The patient was diagnosed with a complicated bite wound. The intervention included surgical debridement, application of an AFSG (Kerecis), negative pressure wound therapy (NPWT), and broad-spectrum antibiotic therapy for a Pasteurella multocida infection. Complete wound closure was achieved within 10 weeks, and the patient had no complications.

**3d Conclusion—What is the main “take-away” lesson(s) from this case:**

The proactive application of a Kerecis graft in an acute, complicated bite wound, especially in a patient with comorbidities known to impair healing, can accelerate wound closure and prevent complications.

**4. Introduction**

The use of cellular, acellular, and matrix-like products (CAMPs) has shown promising results in treating complex chronic wounds like diabetic foot ulcers. However, there is a lack of high-level clinical evidence, such as randomized controlled trials, for their use in acute, contaminated wounds like animal bites. While some products, including Kerecis, mention "animal bites" as a potential application, this is not supported by specific study data. The rationale for using Kerecis in a contaminated wound is based on its reported antimicrobial and anti-inflammatory properties, which can help create an optimal healing environment. The polymicrobial nature of bite wounds, with the predominant pathogen being Pasteurella multocida, and the high risk of rapid, deep tissue infection, present a significant challenge, especially in patients with comorbidities like diabetes or rheumatoid arthritis that compromise wound healing. Early and aggressive management, including surgical debridement and appropriate antibiotic therapy, is crucial to prevent chronic infection and prolonged wound healing.

**Patient Information**

**5a De-identified patient specific information:** A 54-year-old female patient.

**5b Primary concerns and symptoms:** The patient presented immediately after sustaining a dog bite injury, with an open wound and a large skin and subcutaneous defect on the distal-lateral aspect of her right lower leg.

**5c Medical, family, and psycho-social history**: The patient’s medical history is significant for rheumatoid arthritis (managed with corticosteroids) and type 2 diabetes mellitus. These comorbidities are well-known to cause delayed wound healing.

**5d Relevant past interventions with outcomes:** The patient has a history of prolonged wound healing and scar tissue formation after previous injuries.

**6. Clinical Findings:**

Initial clinical assessment showed no signs of an active infection. The patient’s laboratory infection parameters were all within the normal range at the time of admission.

**7. Timeline**

September 28, 2024: The patient sustained a dog bite injury and presented immediately. A culture swab was taken. The patient underwent surgical debridement of the wound on the same day, followed by the application of a Kerecis fish skin graft. NPWT was applied, and intravenous amoxicillin-clavulanic acid was started.

Day 3 Post-op: The NPWT dressing was removed. The wound was assessed, showing a partially integrated Kerecis graft with beginning granulation and no signs of infection. The patient was discharged with an oral course of amoxicillin-clavulanic acid.

Weeks 4, 6, 8, 10: Regular follow-up and wound care were performed. The wound showed continuous progress in healing, with good granulation and epithelialization.

Week 10: The wound achieved complete closure.

6-Month Follow-up: The wound remained fully closed, and the quality of the new skin and scar tissue was clinically assessed as excellent, with good elasticity.

**Diagnostic Assessment**

**8a Diagnostic testing:**

A culture swab was taken from the wound on admission. Laboratory tests for infection parameters were normal. The culture results later isolated Pasteurella multocida, which was sensitive to the prescribed antibiotics.

**8b Diagnostic challenges:**

No significant diagnostic challenges were encountered.

**8c Diagnosis:**

The diagnosis was a complicated dog bite wound with deep skin and soft tissue defect.

**8d Prognosis:**

Given the patient's comorbidities and the nature of the wound, the prognosis for spontaneous healing was poor, with a high risk of prolonged healing and the wound becoming chronic without interventation.

**Therapeutic Intervention**

**9a Types of therapeutic intervention:**

The patient underwent surgical debridement to remove all devitalized tissue. This was followed by the application of a Kerecis fish skin xenograft, which was secured with sutures. NPWT was applied to the wound bed to optimize healing, and the patient was treated with intravenous and then oral amoxicillin-clavulanic acid.

**9b Administration of therapeutic intervention:**

The Kerecis graft was sutured to the wound edges. NPWT was applied at a continuous pressure of 125 mmHg. Amoxicillin-clavulanic acid was administered intravenously initially and then as a 1g oral dose twice daily for 7 days.

**9c Changes in therapeutic intervention:**

There were no changes in the therapeutic intervention.

**Follow-up and Outcomes**

**10a Clinician and patient-assessed outcomes:**

The wound healed completely within 10 weeks. The patient reported no pain starting on the third day after surgery and was able to return to work as a school teacher after one week. The scar quality at the 6-month follow-up was noted to be clinically superior and more elastic than the surrounding parchment-like skin.

**10b Important follow-up diagnostic and other test results:**

The microbiology results confirmed that the Pasteurella multocida infection was sensitive to the administered amoxicillin-clavulanic acid, justifying the chosen antibiotic regimen.

**10c Intervention adherence and tolerability:**

The patient demonstrated excellent adherence to the prescribed oral antibiotics and the wound care regimen. The treatment was well-tolerated, as evidenced by the patient's swift return to normal activities.

**10d Adverse and unanticipated events:**

No adverse or unanticipated events occurred.

**Discussion**

**11a A scientific discussion of the strengths AND limitations associated with this case report**:

This case report highlights the successful use of an AFSG in an under-documented indication and provides a detailed account of the management of a complicated bite wound in a high-risk patient. It offers valuable insights into a potential treatment option where high-level evidence is lacking. The primary limitation is that it is a single case report, and the findings cannot be generalized to a broader patient population. The patient's positive outcome is a result of a combination of interventions (debridement, antibiotics, NPWT, and the graft), making it difficult to isolate the precise contribution of the Kerecis graft alone.

**11b Discussion of the relevant medical literature with references:**

The case aligns with existing literature on the challenges of treating complicated bite wounds, particularly in patients with comorbidities like diabetes and rheumatoid arthritis, which can significantly prolong healing. The successful outcome supports the proposed antimicrobial and anti-inflammatory properties of AFSGs, which help to create a better healing environment, especially in contaminated wounds. The prompt identification and treatment of the Pasteurella multocida infection, as highlighted in the literature, were also crucial to the success of this case. A similar successful outcome was reported in a veterinary case study using a different type of fish skin graft for a dog bite wound, further supporting the potential application of these grafts in this specific context.

**References**

1. Lee YJ, Han HJ, Shim HS. Treatment of hard-to-heal wounds in ischaemic lower extremities with a novel fish skin-derived matrix. J Wound Care. 2024 May 2;33(5):348–56. doi: 10.12968/jowc.2024.33.5.348.

2. Karhana S, Khan MA. Omega-3 Acellular Fish Skin Grafts for Chronic and Complicated Wounds: A Systematic Review of Efficacy and Safety. Dermatol Pract Concept. 2025 Apr 1;15(2):4945. doi: 10.5826/dpc.1502a4945.

3. Dardari D, Potier L, Sultan A, François M, M'bemba J, et al. Intact fish skin graft vs. standard of care in patients with neuroischaemic diabetic foot ulcers (KereFish Study): An international, multicentre, double-blind, randomised, controlled trial study design and rationale. Medicina. 2022;58(12):1775. doi: 10.3390/medicina58121775.

4. The marine Omega3 wound matrix for treatment of complicated wounds: a multicenter experience report. Dorweiler B, Trinh TT, Dünschede F, Vahl CF, Debus ES, Storck M, Diener H. Gefasschirurgie. 2018;23:46–55. doi: 10.1007/s00772-018-0428-2.

5. Accelerated wound closure of deep partial thickness burns with acellular fish skin graft. Stone R 2nd, Saathoff EC, Larson DA, et al. Int J Mol Sci. 2021;22:1590. doi: 10.3390/ijms22041590.

6. *Necrotizing fasciitis: a fourteen-year retrospective study of 163 consecutive patients. Childers BJ, Potyondy LD, Nachreiner R, et al.*<https://pubmed.ncbi.nlm.nih.gov/11842952/>*Am Surg. 2002;68:109–116.*

7. Oracle Medical Advisory Board & Editors. Guideline Directed Topic Overview. 2025.

8. Giordano A, Dincman T, Clyburn BE, Steed LL, Rockey DC. Clinical features and outcomes of Pasteurella multocida infection. Medicine (Baltimore). 2015 Sep 11;94(36):e1285. doi: 10.1097/MD.0000000000001285.

9. Boadu C, Hernandez A, Zeidan B Jr, Young JT, Frunzi J. Pasteurella multocida bacteremia in an immunocompromised patient after multiple cat scratches. Cureus. 2021 Jan 27;13(1):e12938. doi:10.7759/cureus.12938.

10. <https://www.kerecis.com/fish-skin-technology/>

11. *Chiang A.D., Zurlo J.J. In: Principles and practice of infectious diseases. Mandell G.L., Bennett J.E., Dolin R., editors. Elsevier; Philadelphia, PA: 2020. Pasteurella species; pp. 2774–2778.*

*12.* Naas T., Benaoudia F., Lebrun L., Nordmann P. Molecular identification of TEM-1 β-lactamase in a Pasteurella multocida isolate of human origin. Eur J Clin Microbiol Infect Dis. 2001;20:210–213. doi: 10.1007/pl00011254.

13. *Lion C., Lozniewski A., Rosner V., Weber M. Lung abscess due to b-lactamase–producing Pasteurella multocida. Clin Infect Dis. 1999;29:1345–1346. doi: 10.1086/313439.*

14. Cross SL. Pasteurella multocida infection medication; Medscape; Tools & Reference>Infectious Diseases; Updated Jan 02, 2025;

15. Stedman TL. Wound. In: Stedman’s medical dictionary. Philadelphia (PA): Lippincott Williams & Wilkins; 2011.

16. Baranoski S, Ayello EA, Langemo D. Acute and chronic wound healing. In: Wound care essentials: practice principles. 4th ed. Philadelphia (PA): Wolters Kluwer Publishing; 2016. p. 61-78.

17. Chapin JC, Hajjar KA. Fibrinolysis and the control of blood coagulation. Blood Rev. 2015;29(1):17–24. doi: 10.1016/j.blre.2014.07.002.

18. Hess TC. Checklist for factors affecting wound healing. Adv Skin Wound Care [Internet]. 2011 [cited 2018 Apr 16];24(4):192.

19. Cho SK, Mattke S, Sheridan M, Ennis W. Association of wound healing with quality and continuity of care and sociodemographic characteristics. Am J Manag Care. 2022;28(4):e146–e152. doi:10.37765/ajmc.2022.88868

20. Choi C, Linder T, Kirby A, Rosenkrantz W, Mueller M. Use of a tilapia skin xenograft for management of a large bite wound in a dog. Can Vet J. 2021 Oct;62(10):1071–6.

**11c The scientific rationale for any conclusions:**

The rationale for the successful outcome is multifactorial. The Kerecis graft's porous structure prevented fluid collection and potential abscess formation. Its reported anti-inflammatory and antimicrobial properties likely contributed to creating a favorable environment for healing. The timely surgical debridement and targeted antibiotic therapy were essential in controlling the infection. In a patient with comorbidities that impair healing, the proactive use of the graft appears to have accelerated the natural healing process, leading to a much faster wound closure than would be expected with standard care.

**11c The primary “take-away” lessons of this case report:**

Using the Kerecis graft as a proactive measure, even in primary bite wounds without immediate signs of infection, is a key takeaway. This approach aims to prevent complications rather than treating them after they occur. The graft's ability to create an optimal healing environment is particularly valuable for patients with underlying conditions like diabetes mellitus or rheumatoid arthritis, which often lead to delayed healing. The findings of this case should encourage further research. A controlled study comparing outcomes of traditional wound care with Kerecis grafts in similar patient populations would provide more robust evidence and help establish it as a standard of care.

**12. Patient Perspective:** No specific patient perspective was included in the provided manuscript.

**13. Informed Consent:** Written informed consent was obtained from the patient for the publication of this case report and any accompanying images. A copy of the written consent is available upon request.
